# Structural plasticity and associative memory in balanced neural networks with spike-time dependent inhibitory plasticity

**DOI:** 10.1186/1471-2202-16-S1-P235

**Published:** 2015-12-18

**Authors:** Ankur Sinha, Neil Davey, Roderick Adams, Volker Steuber

**Affiliations:** 1 Science and Technology Research Institute, University of Hertfordshire, Hatfield, UK

## 

In recent work, Vogels and collaborators demonstrated the ability of spike-time dependent inhibitory plasticity to stabilise recurrent spiking neural networks by balancing out the excitatory input received by neurons in the network with the required amount of inhibition [1]. Further, as an application of this unsupervised balance, they showed that such a network can operate as a non-attractor associative memory, by being able to store patterns that become indistinguishable from the background activity of the network, but that can be recalled successfully by external stimuli.

In other research, also related to homoeostasis in neuronal networks, Butz and colleagues investigated the rewiring of deafferentated neurons driven by homoeostatic structural plasticity [2,3]. Their work, based on biological observations of restructuring in the visual cortex [4,5], suggests that neurons endeavour to maintain an appropriate level of electrical activity by adjusting the number of synaptic contacts they make. In order to remedy the loss in electrical activity experienced by neurons as a result of deafferentation, they implemented a growth rule that enabled the neurons to increase the number of lateral connections.

In our research, we measure the memory capacity of balanced spiking neural networks possessing capabilities of both fast synaptic and slow structural plasticity by calculating a signal to noise ratio [6]. The memory capacity of a network of neurons is dependent on various parameters such as the magnitude of the recall stimulus, the size of the pattern relative to the size of the network, the learning rule that dictates inhibitory plasticity, and so on. In this work, as an initial step, we investigate the memory capacity of a non lesioned network containing spike-time dependent plastic inhibitory synapses similar to the network used by Vogels and collaborators. Random, possibly overlapping patterns are iteratively stored in the network as Hebbian assemblies. In order to measure their signal to noise ratio, the stored patterns are recalled by the activation of a randomly selected subset of their neurons. In general, during the recall of the stored patterns, we observe a gradual decrease in the signal to noise ratio as more and more patterns are stored in the network. This signifies an inverse relation between the mean signal to noise ratio and the total number of such patterns that were stored in the network. We are currently investigating the performance of our balanced networks during and after focal lesions and homoeostatic rewiring.
